# NF-κB p65-dependent transcriptional regulation of histone deacetylase 2 contributes to the chronic constriction injury-induced neuropathic pain via the microRNA-183/TXNIP/NLRP3 axis

**DOI:** 10.1186/s12974-020-01901-6

**Published:** 2020-07-28

**Authors:** Jiamin Miao, Xuelong Zhou, Tianjiao Ji, Gang Chen

**Affiliations:** 1 Department of Anesthesiology, Sir Run Run Shaw Hospital, Zhejiang University School of Medicine, No. 3, Qingchun East Road, Jianggan District, Hangzhou, 310012 Zhejiang Province China; 2 Department of Anesthesiology, The First Affiliated Hospital, College of Medicine, Zhejiang University, Hangzhou, 310003 China; 3 Laboratory for Biomaterials and Drug Delivery, Department of Anesthesiology, Boston Children’s Hospital, Harvard Medical School, Boston, 02115 USA

**Keywords:** Neuropathic pain, Chronic constriction injury, NF-κB p65, Histone deacetylase 2, microRNA-183, TXNIP, NLRP3

## Abstract

**Background:**

Neuropathic pain is related to the sustained activation of neuroglial cells and the production of proinflammatory cytokines in the spinal dorsal horn. However, the clinical efficacy of currently available treatments is very limited. The transcription factor nuclear factor κB (NF-κB) is a ubiquitously expressed protein family and considered to be crucial in autoimmunity. Thus, our study aimed to examine the influence of NF-κB p65 in chronic constriction injury (CCI)-induced neuropathic pain as well as its underlying mechanism.

**Methods:**

A rat model of neuropathic pain was established by CCI induction followed by isolation of microglial cells. The binding of NF-κB p65 to HDAC2, of miR-183 to TXNIP, and of TXNIP to NLRP3 was investigated. Expression of miR-183, NF-κB p65, HDAC2, TXNIP, and NLRP3 was determined with their functions in CCI rats and microglial cells analyzed by gain- and loss-of-function experiments.

**Results:**

NF-κB p65 and HDAC2 were upregulated while miR-183 was downregulated in the dorsal horn of the CCI rat spinal cord. NF-κB p65 was bound to the HDAC2 promoter and then increased its expression. HDAC2 reduced miR-183 expression by deacetylation of histone H4. Additionally, miR-183 negatively regulated TXNIP. Mechanistically, NF-κB p65 downregulated the miR-183 expression via the upregulation of HDAC2 and further induced inflammatory response by activating the TXNIP-NLRP3 inflammasome axis, thus aggravating the neuropathic pain in CCI rats and microglial cells.

**Conclusion:**

These results revealed a novel transcriptional mechanism of interplay between NF-κB and HDAC2 focusing on neuropathic pain via the miR-183/TXNIP/NLRP3 axis.

## Background

Neuropathic pain is a universal disease in clinical practice, with the prevalence ranging from 7 to 10% in the general population [1]. The most common causes of neuropathic pain include inflammation-induced central and peripheral neuron sensitization in the spinal dorsal horn [2]. Patients with neuropathic pain exhibit frequent and severe depression, sleep disturbances, and anxiety, which further enhance the burden on their life quality [3]. However, due to the complex pathogenesis of neuropathic pain, effective therapy remains a great challenge [4]. Chronic constriction injury (CCI) is a widely used model to induce neuropathic pain in experimental animals [5]. Thus, it is urgently required to explore the molecular mechanism and develop innovative targets to relieve CCI-induced neuropathic pain.

Accumulating data have demonstrated that nuclear factor-kappa B (NF-κB), which transfers receptor-mediated signals to the nucleus to regulate the pro- and antinociceptive factors, is implicated in the process of neuropathic pain [6]. Accordingly, Yin et al. have elucidated that NF-κB inhibitor (pyrrolidine dithiocarbamate, PDTC) plays a suppressive role in neuropathic pain [7]. Moreover, NF-κB is regarded as a crucial regulator of histone deacetylase 2 (HDAC2) [8], while histone deacetylase 2 (HDAC2), a member of HDAC, is reported to be involved in neuropathic pain [9]. Importantly, HDAC2 exhibits significant effects on the progression of dorsal horn following peripheral injury [10]. A previous study has revealed that the depletion of HDAC could regulate the microRNA-183 (miR-183) to affect the tumor process [11]. Also, previously reported studies have verified the impact of miR-183 in the regulation of CCI-induced neuropathic pain [12]. Nevertheless, our results from microarray analysis further verified that miR-183 could affect the neuropathic pain by regulating the thioredoxin interacting protein (TXNIP)/NLR family pyrin domain-containing 3 (NLRP3) inflammasome axis in peripheral nerve injury. TXNIP (also known as thioredoxin binding protein-2 or vitamin D3 upregulated binding protein-2) is considered as a key regulator for the formation of the NLRP3 inflammasome [13], whilst the elevation of NLRP3 inflammasome has been proved to aggravate the process of neuropathic pain [14]. Yet, the specific mechanisms of NF-κB p65-mediating HDAC2 to regulate the miR-183/TXNIP/NLRP3 axis in neuropathic pain remain poorly understood. Thus, in the present study, CCI models were established to explore the role of NF-κB p65-mediating HDAC2 inflammatory response in neuropathic pain through the miR-183/TXNIP/NLRP3 axis along with its possible mechanisms, which may help to provide a novel direction for treating neuropathic pain.

## Materials and methods

### Ethics statement

Animal experiments were approved by the Ethics Committees of Zhejiang University School of Medicine and conducted by following the Guide for the Care and Use of Laboratory Animals published by the US National Institutes of Health. All efforts were made to minimize the number and suffering of the included animals.

### CCI rat model establishment

Sprague-Dawley (SD) male rats weighing 180–200 g (purchased from SLAC Laboratory Animal Co. Ltd. Shanghai, China) were used in this study. After adaptive feeding for 1 week, the rats were given a normal diet and drinking water, after which they were randomly divided into the normal control group, sham group, and CCI group (10 rats in each group). The CCI rat model was established as previously described [2]. Briefly, the rats were anesthetized by intraperitoneal injection of 3% pentobarbital sodium at a dose of 40 mg/kg, and the sciatic nerve of rat right thigh was exposed by blunt dissection. Proximal to the sciatic nerve, about 7 mm of the nerve without adhering tissue and four ligatures (about 1 mm interval) were ligated around the sciatic nerve until a brief twitch was observed. The rats in the sham group were subjected to the same procedures as mentioned before except for the ligation. The surgical incision was disinfected with iodine, and the wound was sutured.

### Animal treatment

The CCI rats (14 days of CCI induction) were treated with 50 mg/kg NF-κB p65 inhibitor PDTC (once a day for 14 days via intraperitoneal injection), lentiviral vectors containing overexpression (oe)-HDAC2 (injected once via tail vein), 20 mg/kg SAHA (HDAC inhibitor, once a day for 14 days via intraperitoneal injection), lentiviral vectors containing short hairpin (sh)-HDAC2 (injected once via tail vein), miR-183 agomir (injected once via tail vein), and lentiviral vectors containing oe-TXNIP (injected once via tail vein). The scrambled shRNA vector and agomir negative control (NC) were served as NCs. NF-κB p65 inhibitor PDTC (Beyotime Institute of Biotechnology, Shanghai, China) and SAHA inhibitor (Sigma-Aldrich Chemical Company, St Louis, MO, USA) were dissolved in the 5% dimethyl sulfoxide (DMSO), respectively, sub-packed, and preserved at – 20 °C. After 14 days, the rats were euthanized after anesthesia using 5% pentobarbital sodium (30 mg/kg) (P3761, Sigma-Aldrich Chemical Company, St Louis, MO, USA). The dorsal horn of the spinal cord of rats was isolated and collected for detecting the expression of related genes.

### Cell culture and microglial cell isolation

Human embryo kidney (HEK293T) cells (American Type Culture Collection [ATCC], Manassas, VA, USA, CRL-1573) were grown in Dulbecco’s modified Eagle’s medium (DMEM) containing 10% fetal bovine serum (FBS), and cultured in a 5% CO_2_ incubator at 37 °C. Microglial cells were isolated from the dorsal horn of the spinal cord in SD rats and cultured according to the previously reported method [15]. Microglial cells were added with DMEM/F12 (Gibco, Carlsbad, California, USA) containing 10% FBS (Hyclone Laboratories, Logan, UT, USA), 100 U/mL penicillin, and 100 mg/mL streptomycin and then cultured in a flask (75 cm^2^) for 14 days. At day 10, microglial cells were isolated from primary mixed glial cell culture by shaking on a rotary oscillator at 300 rpm at 37 °C overnight. After 2 days, the CD11b antibody (#31-1174-00, BD Biosciences, Franklin Lakes, NJ, USA) was adopted to measure the purity of primary microglial cells, which was over 95% of purity.

### Construction and packaging of lentiviral vectors

The lentivirus packaging system was constructed via LV5-GFP (lentivirus gene overexpression vector) and pSIH1-H1-copGFP (lentivirus gene silencing vector). HDAC2 shRNA, NF-κB p65 shRNA, miR-183 mimic, mimic NC, and scramble shRNA were provided by Shanghai GenePharma Co. Ltd. (Shanghai, China). The packaging lentivirus and target vector were co-transfected into the HEK293T cells and cultured for 48 h. The supernatant was then collected, and the lentivirus particles in the supernatant were filtered to detect virus titer. Then, the lentiviruses in the exponential phase were collected, and the cells were infected with lentiviruses containing sh-NC, sh-HDAC2, oe-NC, oe-TXNIP, mimic NC, miR-183 mimic, and miR-183 mimic + oe-TXNIP, respectively. When the cells grew to the logarithmic growth phase, they were trypsinized and mechanically dissociated into cell suspension (5 × 10^4^ cells/mL) which was then seeded into 6-well plates (2 mL/well) and incubated at 37 °C overnight. After 48 h of transfection, reverse transcription-quantitative polymerase chain reaction (RT-qPCR) was used to detect the expression of related genes in cells.

### Intrathecal catheters and drug treatment

For spinal drug administration, all male SD rats were implanted with intrathecal catheters by inserting them into the subarachnoid space between the L5 and L6 vertebrae. After surgical procedures, the rats were housed individually and had free access to water and food which was provided. On the 2nd day after surgical procedure, the rats were injected with 2% lidocaine (30 μL) through catheters over 30 s followed by a 10-μL flush of normal saline. Hind paw paralysis and/or paresis within 30 s and lasting 6–10 min indicated a successful catheterization. Rats exhibiting postoperative neurologic deficits (about 25%) were excluded. The shRNA corresponding to NF-κB p65, HDAC, TXNIP, and NC was cloned into a pFU-GW-RNAi-GFP vector (Shanghai Gene Chem Co., Ltd., Shanghai, China). To determine the level of infection efficiency of lentivirus constructs, LV-GFP was preliminarily studied. Then, the lentivirus was injected into the rats, and the experiments were conducted four weeks later.

### Neuropathic pain behavior test

Mechanical allodynia and thermal hyperalgesia were measured to assess the response of rats to neuropathic pain [2]. Behavior test was carried out at 1 day before the surgical procedure at days 1, 3, 5, 7, and 14, respectively, after sciatic nerve injury in the rat right thigh. Rats were placed individually in a wire mesh cage and habituated for 30–60 min. Calibrated von Frey filaments in the mesh cage were applied to the plantar surface of the rat hind paw from below the mesh floor. Thermal punctuate stimuli were transferred to the plantar surface of the hind paw with a focused beam of radiant heat, and the withdrawal latency time was recorded. The 50% mechanical paw withdrawal threshold (PWT) was determined using the up-down method. Thermal hyperalgesia was assessed by measuring the latency of paw withdrawal in response to a radiant heat source. Thermal sensitivity was determined by the thermal paw withdrawal latency (PWL). The result of each test was expressed as the mean of three withdrawal latencies in seconds. Rats were acclimated to the test environment at least 30 min before the commencement of the experiments. Behavioral analysis was performed between 09: 00 and 12:00 in a quiet room.

### Dual-luciferase reporter assay

The 3′untranslated region (3′UTR) of TXNIP binding with miR-183 was inserted into the dual-luciferase reporter gene vector pmirGLO. Site-directed mutagenesis of wild-type (WT) reporter gene plasmid pmirGLO-TXNIP-WT was carried out using a mutation kit. Then, the reporter plasmids were co-transfected with miR-183 mimic and NC mimic (both were purchased from Shanghai GenePharma Co. Ltd., Shanghai, China) into 293 T cells. After transfection for 24 h, the cells were lysed at room temperature for 15 min to obtain the supernatant. The luciferase activity was measured using a dual-Luciferase Reporter Assay System (E1910, Promega, Madison, WI, USA). Thereafter, 20 μL samples were added with 100 μL firefly luciferase and 100 μL Renilla luciferase working solution to detect the activity of firefly luciferase and Renilla luciferase, respectively. The ratio of firefly luciferase activity to internal Renilla luciferase activity was regarded as the relative luciferase activity. Each sample was repeated more than 3 times.

### Western blot analysis

Following 14 days of CCI construction, the tissue samples of the dorsal horn of rat spinal cord were washed with pre-cooled phosphate-buffered saline (PBS), lysed with radioimmunoprecipitation assay (RIPA) lysis buffer (C0481, Sigma-Aldrich Chemical Company, St Louis, MO, USA) on ice for 30 min, and centrifuged at 12000*g* and 4 °C for 15 min. The supernatant was then collected, and the protein concentration was measured using the bicinchoninic acid (BCA) protein concentration test kit (Beyotime). A total of 20 μg protein samples were subjected to separation by 10% sodium dodecyl sulfate-polyacrylamide gel electrophoresis (SDS-PAGE) and transferred onto a polyvinylidene fluoride (PVDF) membrane (Millipore, Billerica, MA, USA) at a constant current of 250 mA. The membranes were blocked with 5% skimmed milk powder at ambient temperature for 1 h followed by overnight incubation at 4 °C with primary rabbit antibodies to NF-κB phosphorylated (p)-p65 (#3037, 1:500, Cell Signaling Technology), NF-κB P65 (ab16502, 1:1000, Abcam, Cambridge, UK), HDAC2 (ab16032, 1: 500, Abcam), TXNIP (ab188865, 1:1000, Abcam), NLRP3 (ab232401, 1: 1000, Abcam), apoptosis-associated Speck-like protein containing a CARD (ASC; ab47092, 1: 1000, Abcam), caspase-1 (ab179515, 1: 1000, Abcam), and glyceraldehyde-3-phosphate dehydrogenase (GAPDH; ab181602, 1: 1000, Abcam). The next day, the membrane was incubated with horseradish peroxidase-labeled secondary antibody to immunoglobulin G (IgG) (ab99702, 1:1000, Abcam) for 1 h at room temperature. Thereafter, the membrane was developed using enhanced chemiluminescence (Shanghai Baoman Biotechnology Co., Ltd., Shanghai, China), and the gray value was analyzed using the Image J software. The protein level was represented by the ratio of the gray value of target bands to that of the internal reference (GAPDH).

### RNA isolation and quantitation

The expression of NF-κB p65, HDAC2, TXNIP, NLRP3, and miR-183 in the dorsal horn of rat spinal cord and microglial cells was determined using the RT-qPCR. Dorsal horn of spinal cord and microglial cells was lysed using the TRIzol reagent (Invitrogen, Carlsbad, California, USA) followed by total RNA extraction with phenol-chloroform at room temperature for 10–30 min. The purity (260/280 = 1.8–2.0) and concentration of the extracted RNA were then determined by a nucleic acid quantitative instrument. Subsequently, 400 ng of the extracted RNA was transcribed into complementary DNA (cDNA) using the PrimeScript RT Reagent Kit (Takara Bio Inc., Otsu, Shiga, Japan). RT-qPCR was conducted using the chimeric dye SYBR® Premix Ex Taq^TM^ II kit (Takara Bio Inc., Otsu, Shiga, Japan). Amplification was conducted using the Thermal Cycler Dice Real-Time System (TP800, Takara). The primers for RT-qPCR were synthesized by Guangzhou RiboBio Co., Ltd. (Guangzhou, China) (Table 1) while the primers for miR-183 were designed using the stem-loop method [16]. With GAPDH regarded as the internal reference of mRNAs and U6 of miRNA, the expression of the target gene was calculated using the 2^-ΔΔCt^ method [16]. The experiment was repeated at least 3 times independently.

**Table 1:** Primer sequences for RT-qPCR

Target	Primer sequences (5′-3′)
NF-κB p65	F: 5′-GCCTGACACCAGCATTTGA-3′
	R: 5′-CAAACCAAACAGCCTCACG-3′
HDAC2	F: 5′-TGACATTGTGCTTGCTGTCC-3′
	R: 5′-CCCTCAAGTCTCCTGTTCCA-3′
TXNIP	F: 5′-AAGCGTTGAGTAGTACAGATGAG-3′
	R: 5′-GGTATGGCGTGGCA GAGTC-3′
NLRP3	F: 5′-AGCCTCAGGGCA CCA AA-3′
	R: 5′-GGTATGGCGTGGCAAGAGTC-3′
miR-183	F: 5′-TATGGCACTGGTAGAATTCACT-3′
U6	F: 5′-CTCGCTTCGGCAGCACA-3′
	R: 5′-AACGCTTCACGAATTTGCGT-3′
GAPDH	F: 5′-GCCTGACACCAGCATTTGA-3’
	R: 5′-CAAACCAAACAGCCTCACG-3’

### Immunofluorescence staining

Following 14 days of CCI model construction, the transverse spinal cord of rats was cut into 25-μm-thick cryostat sections. Afterward, the tissue sections were soaked twice in PBS, fixed with 4% paraformaldehyde at room temperature for 15 min, washed twice with PBS, treated with 0.5% Triton X-100 for 10 min, and washed twice with PBS. Then, the sections were incubated with normal donkey serum (#017-000-121, Jackson ImmunoResearch, West Grove, PA, USA) at room temperature for 2 h. Subsequently, the sections were incubated with primary rabbit antibodies to NF-κB p-p65 (ab16502, 1:200, Abcam), HDAC2 (ab32117, 1:200, Abcam), TXNIP (1:50; sc-271238, Santa Cruz Biotechnology, Santa Cruz, CA, USA), NLRP3 (ab4207, 1:100, Abcam), and goat-anti Iba-1 polyclonal antibody (ab5076, 1: 500, Abcam) at 4 °C for 24 h. The next day, the sections were re-probed with secondary antibodies to the Alexa Fluor 647 (ab15007, 1: 500, Abcam) or the Alexa Fluor 488 (ab150137, 1:500, Abcam) in the dark at 37 °C for 2 h. The nucleus was stained using 5 μg/mL DAPI at room temperature for 5 min, and the fluorescence decay-resistant medium was added for blocking. The image was observed under an inverted fluorescence microscope (FV1000, Olympus Corp., Tokyo, Japan). The same part of the fluorescence image was selected, and the Image J software was used to analyze the superimposed integrated optical density (IOD), with more than 5 samples in each group. The expression of NF-κB p-p65, HDAC2, TXNIP, and NLRP3 in Iba-1-labeled microglial cells was analyzed using a laser confocal microscopy.

### Enzyme-linked immunosorbent assay (ELISA)

The tissue samples of the spinal dorsal horn of CCI rats were collected after 14 days. The tissue samples were added with 60 μL RIPA cell lysis (Beyotime Institute of Biotechnology, Shanghai, China) and centrifuged at 12000 rpm and 4 °C for 10 min to collect the supernatant. Then, 10 μL samples were obtained to detect protein concentration using the BCA protein assay kit. The subsequent procedures were conducted according to interleukin (IL)-1β ELISA Kit. Then, the optical density (OD) value of each well (96-well plates) was measured at the wavelength of 562 nm using ELISA (Vafioskan Flash, Thermo, USA). The standard curve was drawn with protein concentration as the X-axis and the OD value as the Y-axis. According to the OD value of the sample well to be tested, the IL-1β concentration was calculated according to the standard curve and expressed as pg/mg of protein.

### Co-immunoprecipitation (Co-IP) assay

IP assay was conducted according to the instructions of Catch and Release Reversible Immunoprecipitation System Kit (Merck Millipore, Billerica, MA, USA). The antibodies used for IP assay were as follows: antibody to TXNIP-1 (sc271238, Santa Cruz Biotechnology, Santa Cruz, CA, USA) and antibody to NLRP3 (ab4207, Abcam). Rabbit/mouse IgG served as an NC for the IP reaction.

### Chromatin immunoprecipitation (ChIP) assay

The ChIP assay was conducted as previously reported [16]. In short, the cultured microglial cells were washed twice with precooled PBS and added with 1% paraformaldehyde for 5 min of crosslinking at room temperature, which was halted with 127 mM glycine. The cells were then lysed in the ChIP-grade cell lysis buffer containing 50 mM Tris HCl (pH 8.1), 1% SDS, 10 mM ethylenediaminetetraacetic acid (EDTA), and complete protease inhibitor mixture (Roche Diagnostics GmbH, Mannheim, Germany) on ice for 30 min. Then, the cells were subjected to ultrasonic treatment to make the DNA fragments (200–1000 bp). The subsequent ChIP assay was conducted using the ChIP Assay Kit (Millipore). Briefly, 1 μg DNA was added with protein A agarose and ChIP-grade anti-ac-H4 (ab15823, Abcam) or anti-HDAC2 (sc-81599, Santa Cruz Biotechnology) for incubation overnight 4 °C. Normal mouse antibody to IgG (sc-2025, Santa Cruz Biotechnology) or normal rabbit antibody to IgG (sc-2027, Santa Cruz Biotechnology) served as NC. The next day, the DNA complex was washed with low-salt and high-salt buffer and treated with protease K at 56 °C for 2 h, after which the DNA was extracted and purified by phenol/chloroform. The primers for specific ChIP-qPCR amplification of miR-183 promoter region were F: 5′-CGTAGGGCCACTGGACGA-3’ and R: 5′-TTGTCCCCATTCCAGCCCTG-3′.

### Statistical analysis

All data analyses were conducted using the SPSS 21.0 software (IBM Corp. Armonk, NY, USA). Measurement data conforming to the normal distribution and homogeneity of variance were expressed as the mean ± standard deviation. Data between the two groups were compared using unpaired *t* test while data among multiple groups were compared by one-way analysis of variance (ANOVA) followed by Tukey’s post hoc test with corrections for multiple comparisons. Data comparisons at different time points were conducted using repeated-measures ANOVA followed by Bonferroni post hoc test. Statistical significance was set at *p* < 0.05.

## Results

### NF-κB p65 and HDAC2 were upregulated in the dorsal horn of CCI rat spinal cord

**Fig. 1:**
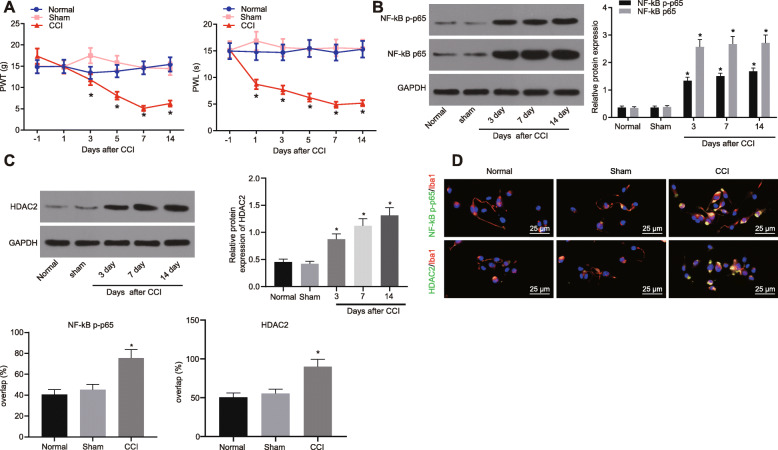
NF-κB p65 and HDAC2 are highly expressed in the dorsal horn of the CCI rat spinal cord. **a** PWT and PWL measured in the dorsal horn of CCI rats (*n* = 10), normal rats (*n* = 10), and sham-operated rats (*n* = 10). **b**, **c** Protein levels of NF-κB p65 and HDAC2 in dorsal horn of CCI rat spinal cord at different time points following CCI modeling determined by Western blot analysis, normalized to GAPDH. **d** Expression of NF-κB p65, NF-κB p-p65, and HDAC2 with Iba-1-labeled microglial cells of CCI rat spinal cord detected by immunofluorescence staining. Iba-1 is a marker molecule of microglial cells. Image J software was used to analyze the IOD (scale bar = 25 μm). *relative to sham-operated or normal rats indicates *p* < 0.05. All measurement data were expressed as the mean ± standard deviation. Unpaired *t* test was used to analyze data between the two groups, one-way ANOVA and Tukey’s post hoc test to analyze data among multiple groups, and repeated-measures ANOVA with Bonferroni post hoc test to analyze data at different time points

CCI rat models were established and assessed by measuring the PWT and PWL. Our results exhibited the decreased PWT on the 3rd day and PWL at the 1st day after the CCI model establishment. However, a remarkable decrease in PWT and PWL was observed at the 5th, 7th, and 14th days post CCI model establishment (Fig. 1a). According to previously reported literature, NF-κB and HDAC play an important role in the regulation of spinal dorsal horn pain; specifically, inhibition of the inflammatory signaling, NF-κB, could alleviate neuropathic pain [2]. The PWT and PWL of animals following CCI model construction can be increased by intrathecal injection of HDAC inhibitor [17]. Thus, in this study, we aimed to explore the expression of NF-κB p65 and HDAC2 and their possible regulatory mechanisms involved in CCI rat models. Western blot analysis was first performed to determine the protein levels of NF-κB p65 and HDAC2 as well as the extent of NF-κB p65 phosphorylation in the dorsal horn of CCI rat spinal cord, and our results exhibited the elevated protein levels of NF-κB p65 and HDAC2 along with the increased extent of NF-κB p65 phosphorylation in dorsal horn at the 3rd, 7th, and 14th days post CCI model establishment (Fig. 1b, c). Furthermore, the expression of HDAC2 and NF-κB p-p65 in Iba-1-labeled microglial cells of the spinal dorsal horn of CCI rats was analyzed by a laser confocal microscope followed by the analysis of the superimposed fluorescence intensity. Our findings revealed that HDAC2 and NF-κB p-p65 were highly expressed in microglial cells of the spinal dorsal horn in CCI rats (Fig. 1d). Thus, it can be speculated that upregulated NF-κB and HDAC2 could regulate neuropathic pain in CCI rats.

### Silencing of NF-κB p65 relieved neuropathic pain in CCI rats by inhibiting HDAC2 expression

**Fig. 2:**
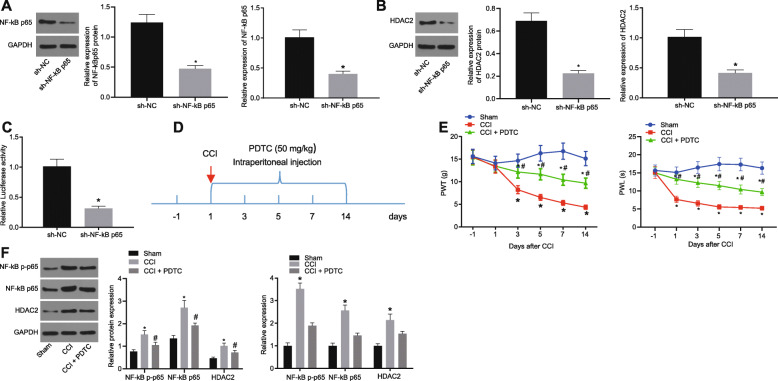
Downregulation of NF-κB p65 attenuates neuropathic pain in CCI rats via inhibition of HDAC2. **a** NF-κB p65 silencing efficiency in microglial cells assessed by Western blot analysis and RT-qPCR. **b** HDAC2 expression upon PDTC treatment determined by Western blot analysis, normalized to GAPDH. **c** The luciferase activity of HDAC2 promoter after NF-κB p65 silencing detected by dual-luciferase reporter assay. **d**, **e** PWT and PWL in CCI rats intraperitoneally injected with PDTC (50 mg/kg, once a day, for 14 days) (*n* = 10). **f** Protein levels of NF-κB p65 and HDAC2 as well as NF-κB p65 phosphorylation extent in spinal dorsal horn in CCI rats determined by Western blot analysis, normalized to GAPDH. *relative to sham-operated rats or microglial cells treated with sh-NC and ^#^relative to CCI rats injected with DMSO indicate *p* < 0.05. All measurement data were expressed as the mean ± standard deviation. Unpaired *t* test was used to analyze data between the two groups, one-way ANOVA and Tukey’s post hoc test to analyze data among multiple groups, and repeated-measures ANOVA with Bonferroni post hoc test to analyze data at different time points. The cell experiment was repeated three times independently

These above-described results revealed the expression pattern of NF-κB p65 and HDAC2 in CCI rats. Thereafter, we aimed to study the underlying mechanism of NF-κB p65 regulating HDAC2 in neuropathic pain. At first, lentivirus-transduced NF-κB p65 knockdown plasmids were constructed, and its silencing efficiency was confirmed to be significant in microglial cells by RT-qPCR and Western blot analysis (Fig. 2a). Meanwhile, treatment with PDTC resulted in a reduction of HDAC2 expression (Fig. 2b). To verify the regulatory relationship between them, we further constructed the HDAC2 promoter-reporter plasmid. The dual-luciferase reporter assay suggested that the luciferase activity of HDAC2 promoter was decreased upon NF-κB p65 knockdown (Fig. 2c). We thus hypothesized that blocking the NF-κB p65/HDAC2 signaling could alleviate the neuropathic pain in CCI rats. To further validate this finding, CCI rats were intraperitoneally injected with NF-κB p65 inhibitor PDTC (50 mg/kg, once a day, for 14 days) to analyze the effect of PDTC on PWT and PWL, our results demonstrated that PWT and PWL were significantly increased, and hyperalgesia was relieved by PDTC (Fig. 2d, e). Additionally, Western blot analysis showed that PDTC remarkably suppressed the protein expression of NF-κB p65 and HDAC2 along with reduced NF-κB p65 phosphorylation extent in the spinal dorsal horn in CCI rats (Fig. 2f). These results suggested that NF-κB p65 silencing repressed the neuropathic pain in CCI rats by downregulating HDAC2.

### Silencing of HDAC2 caused miR-183 upregulation and then relieved neuropathic pain in CCI rats

**Fig. 3:**
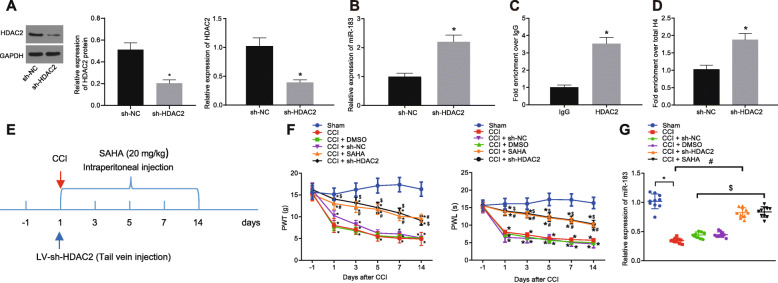
Downregulation of HDAC2 leads to elevated miR-183 expression and attenuates neuropathic pain in CCI rats. **a** HDAC2 silencing efficiency in microglial cells determined by Western blot analysis, normalized to GAPDH. **b** miR-183 expression in microglial cells treated with sh-HDAC2 determined by RT-qPCR, normalized to U6. **c** Enrichment of HDAC2 in the miR-183 promoter region detected by ChIP-qPCR assay. **d** Effect of HDAC2 silencing on histone acetylated H4 in miR-183 promoter detected by ChIP-qPCR assay. **e**, **f** PWT and PWL of CCI rats injected with SAHA (20 mg/kg, once a day) and lentivirus-transduced sh-HDAC2 (*n* = 10). **g** miR-183 expression in spinal dorsal horn of CCI rats injected with SAHA and lentivirus-transduced sh-HDAC2 determined by RT-qPCR, normalized to U6 (*n* = 10). *relative to sham-operated rats, microglial cells treated with sh-NC, or IgG, ^#^relative to CCI rats, and ^$^relative to CCI rats injected with sh-NC indicate *p* < 0.05. All measurement data were expressed as the mean ± standard deviation. Unpaired *t* test was used to analyze data between the two groups, one-way ANOVA and Tukey’s post hoc test to analyze data among multiple groups, and repeated-measures ANOVA and Bonferroni post hoc test to analyze data at different time points. The cell experiment was repeated three times independently

To further explore the molecular mechanism of HDAC2 regulating neuropathic pain in CCI rats, HDAC2 was silenced in microglial cells isolated from CCI rats. Our results from Western blot depicted that lentivirus-transduced sh-HDAC2 reduced the HDAC2 protein levels in microglial cells (Fig. 3a). The miR-183 expression in microglial cells was found to be increased by the lentivirus-transduced sh-HDAC2 (Fig. 3b). Moreover, the ChIP-qPCR assay described that HDAC2 was enriched in the promoter region of miR-183 (Fig. 3c), and HDAC2 silencing elevated the acetylation of histone H4 and then upregulated the miR-183 expression in microglial cells (Fig. 3d), suggesting that HDAC2 regulated the miR-183 transcription via H4 acetylation. To further confirm whether HDAC2 can mediate the regulatory mechanism of neuropathic pain in vivo, CCI rats were injected with HDAC inhibitor SAHA (20 mg/kg, once a day for 14 days) to detect the PWT and PWL. Our results depicted increased PWT and PWL after treatment with SAHA (Fig. 3e). Moreover, CCI rats were injected with lentivirus-transduced sh-HDAC2 via tail vein, and PWT and PWL were measured; the results of which revealed that the silencing of HDAC2 increased the PWT and PWL in CCI rats (Fig. 3 f). The results from RT-qPCR exhibited the reduced miR-183 expression in the spinal dorsal horn of CCI rats and DMSO-treated CCI rats when compared to sham-operated rats while elevated miR-183 expression was found in the spinal dorsal horn of CCI rats injected with SAHA or lentivirus-transduced sh-HDAC2 (Fig. 3g). The abovementioned evidence demonstrated that HDAC2 could downregulate the miR-183 expression while NF-κB p65 modulated the miR-183 transcription expression by regulating the HDAC2 in CCI rats.

### Upregulation of miR-183 attenuated neuropathic pain in CCI rats by inhibiting TXNIP expression

**Fig. 4:**
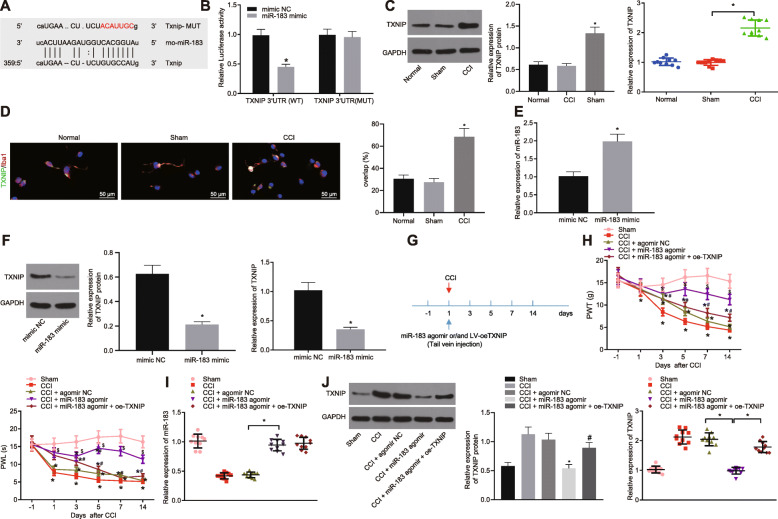
miR-183 relieves neuropathic pain in CCI rats via inhibition of TXNIP. **a** Binding sites between miR-183 and TXNIP predicted by the microrna.org website. **b** Binding relationship between miR-183 and TXNIP detected by dual-luciferase reporter assay (*n* = 3). **c** miR-183 expression and TXNIP mRNA and protein expression in spinal cord of CCI rats determined using RT-qPCR and Western blot analysis, normalized to U6 and GAPDH, respectively (*n* = 10). **d** Expression of TXNIP in Iba-1-labeled microglial cells of CCI rat spinal cord detected by immunofluorescence staining (scale bar = 50 μm) (*n* = 5). **e** miR-183 expression in microglial cells treated with miR-183 mimic determined by RT-qPCR, normalized to U6 (*n* = 3). **f** TXNIP expression in microglial cells treated with miR-183 mimic determined using RT-qPCR and Western blot analysis, normalized to GAPDH (*n* = 3). **g**, **h** PWT and PWL of CCI rats injected with lentivirus expressing miR-183 agomir or in combination with oe-TXNIP following 14 days (*n* = 10). **i** miR-183 expression in spinal cord of CCI rats injected with lentivirus expressing miR-183 agomir or in combination with oe-TXNIP determined by RT-qPCR, normalized to U6 (*n* = 10). **j** TXNIP expression in spinal cord of CCI rats injected with lentivirus expressing miR-183 agomir or in combination with oe-TXNIP determined using RT-qPCR and Western blot analysis, normalized to GAPDH (*n* = 10). *relative to microglial cells treated with mimic NC or sham-operated rats,, ^#^relative to CCI rats, and ^$^relative to CCI rats injected with agomir NC indicate *p* < 0.05. All measurement data were expressed as the mean ± standard deviation. Unpaired *t* test was used to analyze data between the two groups, one-way ANOVA and Tukey’s post hoc test to analyze data among multiple groups, and repeated-measures ANOVA and Bonferroni post hoc test to analyze data at different time points

Bioinformatics analysis showed binding sites between miR-183 and 3′UTR of TXNIP (Fig. 4a), which was further verified by the dual-luciferase reporter assay. As shown in Fig. 4b, elevated miR-183 could inhibit the luciferase activity of TXNIP-WT-3′UTR while it did not affect the luciferase activity of TXNIP-mutant (MUT)-3′UTR, suggesting that miR-183 could target the TXNIP. Additionally, RT-qPCR and Western blot analysis indicated that TXNIP expression was increased while miR-183 expression was reduced in CCI rat models (Fig. 4c). Immunofluorescence staining revealed that TXNIP was upregulated in Iba-1-labeled microglial cells of CCI rats (Fig. 4d). Then, we further verified whether miR-183 regulates TXNIP in microglial cells. For this purpose, microglial cells were first transfected with miR-183 mimic, and RT-qPCR and Western blot analysis were performed. Our results showed that after miR-183 mimic transfection, the expression of miR-183 was significantly increased in microglial cells (Fig. 4e) while the protein level of TXNIP was significantly decreased (Fig. 4f). To further clarify the effect of miR-183 on neuropathic pain in rats, CCI rats were injected with miR-183 agomir and lentivirus-transduced oe-TXNIP via the tail vein. The response of related neuropathic pain in rats was analyzed after 14 days. The results are shown in Fig. 4g, h which revealed that miR-183 agomir triggered an increase in PWT and PWL of CCI rats, which was restored by the co-treatment with miR-183 agomir and oe-TXNIP. The RT-qPCR demonstrated that miR-183 was highly expressed but TXNIP was poorly expressed in the spinal cord of CCI rats injected with miR-183 agomir, which was negated by the dual treatment with miR-183 agomir and oe-TXNIP (Fig. 4i, j). Thus, it can be concluded that miR-183 suppressed the neuropathic pain in CCI rats by decreasing the TXNIP expression.

### Upregulation of miR-183 suppressed inflammatory responses in CCI rats by inhibiting the TXNIP-NLRP3 inflammasome axis

**Fig. 5:**
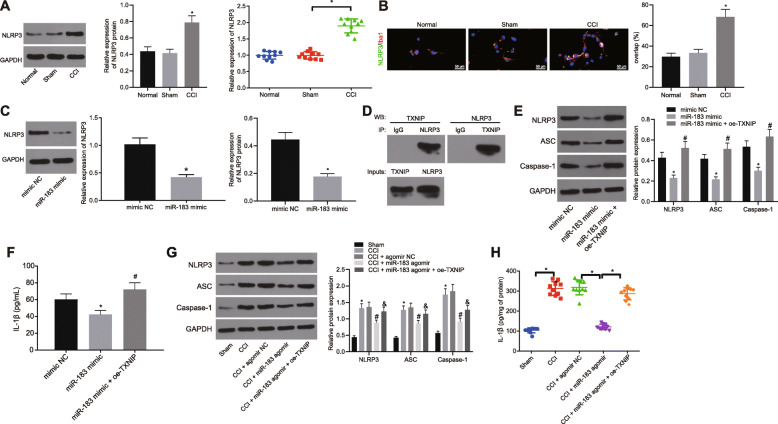
Overexpression of miR-183 inhibits inflammation in CCI rats via the downregulation of the TXNIP-NLRP3 axis. **a** NLRP3 protein expression in the spinal cord of CCI rats determined using Western blot analysis, normalized to GAPDH (*n* = 10). **b** Expression of NLRP3 in Iba-1-labeled microglial cells of CCI rat spinal cord detected by immunofluorescence staining (*n* = 5) (scale bar = 50 μm). **c** NLRP3 protein expression in microglial cells treated with exogenous miR-183 mimicdetermined using Western blot analysis, normalized to GAPDH (*n* = 3). **d** Binding relationship between TXNIP and NLRP3 detected by Co-IP assay. **e** Protein expression of NLRP3, ASC, and caspase-1 in microglial cells after forced expression miR-183 and TXNIP measured using Western blot analysis, normalized to GAPDH. **f** Expression of IL-1β in microglial cells after forced expression miR-183 and TXNIP measured using ELISA (*n* = 10). **g** Expression of NLRP3, ASC, and caspase-1 in spinal cord of CCI rats injected with lentivirus expressing miR-183 agomir or in combination with oe-TXNIP measured using Western blot analysis, normalized to GAPDH. **h** Expression of IL-1β in spinal cord of CCI rats injected with lentivirus expressing miR-183 agomir or in combination with oe-TXNIP measured using ELISA (*n* = 10). *relative to sham-operated rats, microglial cells treated with mimic NC and ^#^relative to microglial cells treated with miR-183 mimic or CCI rats injected with miR-183 agomir indicate *p* < 0.05. All measurement data were expressed as mean ± standard deviation. Unpaired *t* test was used to analyze data between the two groups, one-way ANOVA and Tukey’s post hoc test to analyze data among multiple groups, and repeated measures ANOVA and Bonferroni post hoc test to analyze data at different time points

Subsequently, Western blot analysis was performed to determine the expression of NLRP3 in spinal cord of CCI rats, which exhibited its upregulated expression (Fig. 5a). Our results from immunofluorescence staining revealed that NLRP3 was upregulated in microglial cells (Fig. 5b). The RT-qPCR and Western blot analysis presented that miR-183 mimic reduced the NLRP3 expression in microglial cells from CCI rats (Fig. 5c). To further determine whether TXNIP could activate NLRP3 under neuropathic pain conditions, a Co-IP assay was adopted to detect the binding of TXNIP with NLRP3. Our findings revealed that TXNIP could pull down the NLRP3 protein, whereas NLRP3 could pull down the TXNIP protein (Fig. 5d), indicating that TXNIP could activate the NLRP3 in the process of neuropathic pain. Furthermore, Western blot analysis and ELISA indicated that the protein expression of NLRP3, ASC, caspase-1, and IL-1β was significantly decreased in microglial cells treated with exogenous miR-183 mimic, which was rescued by treatment of oe-TXNIP (Fig. 5e, f). Western blot analysis and ELISA showed that the expression of NLRP3, ASC, caspase-1, and IL-1β was decreased in the spinal cord of CCI rats injected with the lentivirus-expressing miR-183 agomir, which was reversed by the overexpression of TXNIP (Fig. 5g, h). Collectively, the upregulation of miR-183 repressed inflammatory responses in CCI rats via suppression of the TXNIP-NLRP3 inflammasome axis.

### NF-κB p65 promoted neuropathic pain in CCI rats by activating the miR-183-TXNIP-NLRP3 axis via HDAC2

**Fig. 6:**
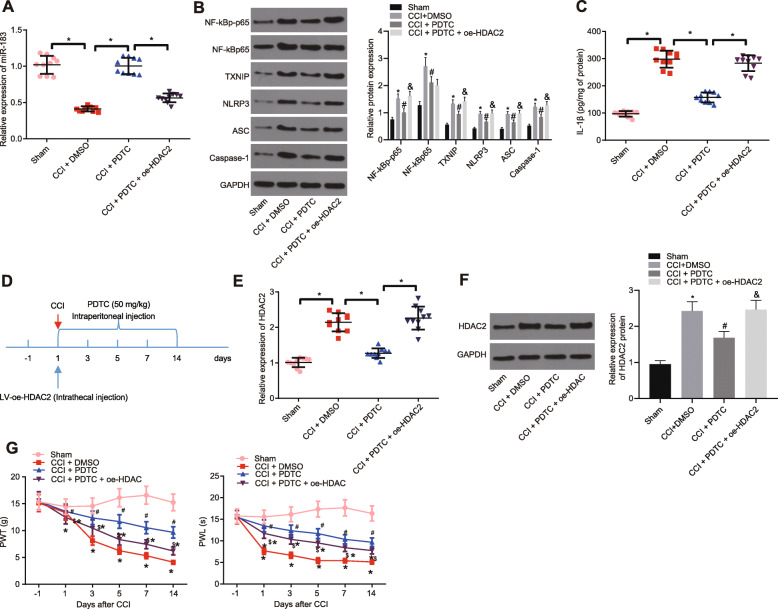
NF-κB p65 facilitated neuropathic pain in CCI rats by affecting the miR-183-TXNIP-NLRP3 axis via HDAC2. CCI rats were injected with lentivirus expressing PDTC or PDTC + oe-HDAC2. **a** miR-183 expression in the spinal cord of CCI rats determined by RT-qPCR, normalized to U6 (*n* = 10). **b** Expression of TXNIP, NLRP3, ASC, and caspase-1 in spinal cord of CCI rats measured using Western blot analysis, normalized to GAPDH. **c** IL-1β expression in spinal cord of CCI rats measured using ELISA (*n* = 10). **d** Molecular level and neuropathic pain-related behavioral changes in rats following 14 days of injection. **e**, **f** HDAC2 expression in spinal cord of CCI rats determined using RT-qPCR and Western blot analysis, normalized to GAPDH (*n* = 10). **g** PWT and PWL of CCI rats were measured. *relative to sham-operated rats, ^#^relative to CCI rats, and ^$^relative to CCI rats injected with PDTC indicate *p* < 0.05. All measurement data were expressed as the mean ± standard deviation. Unpaired *t* test was used to analyze data between the two groups, one-way ANOVA and Tukey’s post hoc test to analyze data among multiple groups, and repeated-measures ANOVA and Bonferroni post hoc test to analyze data at different time points

To further explore the mechanism of miR-183-TXNIP-NLRP3 axis regulated by NF-κB p65 via HDAC2 in neuropathic pain in vivo, the expression of miR-183, TXNIP, NLRP3, and inflammatory corpuscle-related factors, ASC, caspase-1, and IL-1β was measured in the spinal cord of CCI rats treated with NF-κB p65 inhibitor PDTC using the RT-qPCR and Western blot analysis. As documented in Fig. 6a–c, miR-183 expression was elevated in the spinal cord of CCI rats treated with PDTC, while a reduction was observed in the expression of TXNIP, NLRP3, ASC, caspase-1, and IL-1β. Additionally, dual treatment with PDTC and oe-HDAC2 elevated the expression of TXNIP, NLRP3, ASC, caspase-1, and IL-1β in the spinal cord of CCI rats. To further explore whether this mechanism was mediated by the HDAC2, CCI rats treated with PDTC were injected with lentivirus-transduced oe-HDAC2. After 14 days, the molecular level and neuropathic pain-related behavioral changes were compared in rats treated with PDTC and PDTC + oe-HDAC2 (Fig. 6d). Our results from the RT-qPCR and Western blot analyses revealed that the expression of HDAC2 was higher in spinal cord of CCI rats treated with PDTC + oe-HDAC2 than in CCI rats treated with PDTC (Fig. 6e, f). Additionally, compared to PDTC-treated CCI rats, PWT and PWL were increased in CCI rats injected with PDTC + oe-HDAC2 (Fig. 6g). Taken together, NF-κB p65 could downregulate the miR-183 expression in CCI rats through HDAC2 and trigger inflammatory response via activation of the TXNIP-NLRP3 to aggravate neuropathic pain in CCI rats.

## Discussion

Neuropathic pain is a frequently encountered disease that is often resistant to available treatments, exhibiting a poor prognosis in patients [1]. Previously, reported studies have demonstrated the crucial role of NF-κB in the progression of neuropathic pain [7]. Our study aimed to explore the encouraging effect of NF-κB p65-mediating HDAC2 on inflammation in CCI-induced neuropathic pain. Taken together, our study revealed that the silencing of NF-κB p65 significantly reduced the expression of HDAC2, which further inhibits the inflammation and relieves neuropathic pain via the upregulation of miR-183 and downregulation of the TXNIP-NLRP3 inflammasome axis.

Initially, our results demonstrated that in the dorsal horn of CCI rat spinal cord with neuropathic pain, NF-κB p65 and HDAC2 were upregulated, whereas silencing NF-κB p65 suppressed the inflammatory reaction and relieved neuropathic pain in CCI rats by inhibiting the HDAC2 expression. Accordingly, a recently reported study has verified the upregulation of NF-κB during the progression of neuropathic pain while its suppression relieved the neuropathic pain [6]. On the other hand, a well-known NF-κB inhibitor, i.e., PDTC has been reported to possess the potential to repress thermal hyperalgesia and mechanical allodynia in CCI rats [2]. Nevertheless, it is well documented that the main characteristic of neuropathic pain is the activation of inflammatory cytokines, such as IL-1β, which suggests that targeting the inflammatory response could be a crucial factor in treating neuropathic pain [2]. NF-κb (known as a pleiotropic transcriptional factor) exhibits significant impacts in regulating the expression of multiple pro-inflammatory factors such as IL-6, which leads to neuropathic pain [18], whilst inhibition of NF-κB has been proved to attenuate pain and inhibit inflammation after peripheral nerve injury [19]. The p65 subunit of NF-κB has been reported to combine with HDAC2 as part of the same protein complex to negatively regulate the gene transcription in the mouse frontal cortex [20]. Thus, HDAC2 expression is positively related to NF-κB expression [8]. In consent with our findings, HDAC2 expression has been documented to elevate in the superficial dorsal horn of rats [10]. Accordingly, accumulating studies have confirmed the effects of HDAC on the molecular mechanism of neuropathic pain [17]. Importantly, HDAC2 inhibitors have been suggested to ameliorate neuropathic pain by restoring C-fibre sensitivity [21]. Hence, these above-described findings supported the fact that NF-κB p65 knockdown could be considered as a potential therapeutic target to relieve neuropathic pain by suppressing HDAC2.

Furthermore, the data in the current study implied that the depletion of HDAC2 upregulated the miR-183 expression. Consistently, it has been revealed that inhibition of HDAC2 upregulated the miR-183 and exhibits tumor suppressive functions [11]. Moreover, our results from bioinformatics analysis and dual-luciferase reporter gene assay further verified that miR-183 could target TXNIP, whereas overexpression of miR-183 attenuated the neuropathic pain in CCI rats by inhibiting the TXNIP and NLRP3 expression. Recently, aberrant expression of miRNAs has been identified to affect the development and progression of neuropathic pain [22]. On the other hand, miR-183 exhibits poor expression in neuropathic pain while restoration of miR-183 contributes to the attenuation of neuropathic pain [23]. Similarly, Shi et al. have illustrated the reduction of miR-183-5p expression in dorsal root ganglion during the development of CCI-induced neuropathic pain, whereas the upregulation of miR-183-5p remarkably relieved neuropathic pain [12]. Nevertheless, TXNIP has emerged as a potential regulator of the formation of the NLRP3 inflammasome [13], whilst activation of NLRP3 inflammasome has reported contributing to the progression of neuropathic pain, indicating its ability to relieve neuropathic pain upon its inhibition [14]. Moreover, miR-23a has been documented to influence the development of neuropathic pain via regulating the TXNIP/NLRP3 inflammasome axis in spinal glial cells [24]. Hence, we speculated that the upregulation of miR-183 could be attributed to great therapeutic potential in CCI-induced neuropathic pain by affecting inflammation response via inhibition of TXNIP and NLRP3.

## Conclusion

**Fig. 7:**
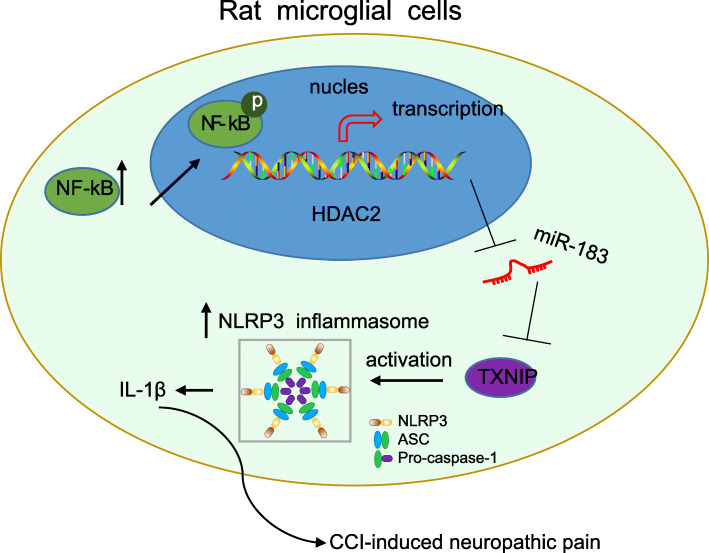
Mechanism graph of NF-κB p65-mediated HDAC2 in CCI-induced neuropathic pain. NF-κB p65-activated HDAC2 could induce inflammation reaction to aggravate neuropathic pain in CCI rats by downregulating miR-183 and promoting TXNIP and NLRP3

In summary, the present study exhibited that NF-κB p65-activated HDAC2 could potentially induce inflammation reaction to aggravate neuropathic pain by downregulating miR-183 and promoting TXNIP and NLRP3 (Fig. 7). Thus, downregulated NF-κB p65-mediating HDAC2 may function as a promising new direction for developing novel treatments for neuropathic pain. However, the research is still in the preclinical stage. Therefore, further investigations are required to unravel the underlying molecular mechanism.

## Data Availability

The datasets generated/analyzed during the current study are available.

## Abbreviations

CCI: Chronic constriction injury
HDAC2: Histone deacetylase 2
NF-κB: Nuclear factor-kappa B
TXNIP: Thioredoxininteracting protein
PDTC: Pyrrolidine dithiocarbamate
miR-183: MicroRNA-183
NLRP3: NLR family pyrin domain containing 3
ATCC: American Type Culture Collection
SD: Sprague-Dawley
oe: Overexpression
sh: Short hairpin
NC: Negative control
DMSO: Dimethyl sulfoxide
DMEM: Dulbecco’s modified Eagle’s medium
FBS: Fetal bovine serum
PWT: Paw withdrawal threshold
PWL: Paw withdrawal latency
WT: Wild type
MUT: Mutant
cDNA: Complementary DNA
RT-qPCR: Reverse transcription quantitative polymerase chain reaction
3′UTR: 3′untranslated region
p: Phosphorylated
GAPDH: Glyceraldehyde-3-phosphate dehydrogenase
IgG: Immunoglobulin G
BCA: Bicinchoninic acid
SDS-PAGE: Sodium dodecyl sulfate-polyacrylamide gel electrophoresis
PVDF: Polyvinylidene fluoride
ECL: Enhanced chemiluminescence
PBS: Phosphate-buffered saline
EDTA: Ethylenediaminetetraacetic acid
ELISA: Enzyme-linked immunosorbent assay
IOD: Integrated optical density
RIPA: Radioimmunoprecipitation assay
IL: Interleukin
OD: Optical density
Co-IP: Co-immunoprecipitation
ChIP: Chromatin immunoprecipitation
ANOVA: Analysis of variance
